# Health-related quality of life in French pediatric patients with X-linked hypophosphatemia: real-world data from the International XLH Registry

**DOI:** 10.1093/jbmrpl/ziaf142

**Published:** 2025-08-30

**Authors:** Agnès Linglart, Cyril Amouroux, Iva Gueorguieva, Jerome Harambat, Jean-Pierre Salles, Diana-Alexandra Ertl, Kerry Sandilands, Angela Rylands, Angela Williams, Haruka Ishii, Annabel Bowden, James W Varni, Justine Bacchetta

**Affiliations:** Université Paris Saclay, AP-HP, INSERM, Service endocrinologie et diabète de l’enfant, Centre de référence des maladies rares du métabolisme du calcium et du phosphate, Hôpital Bicêtre Paris Saclay, 94270 Le Kremlin-Bicêtre, France; Hôpital Arnaud de Villeneuve, 34295 Montpellier, France; CHRU, 59000 Lille, France; Service de Néphrologie Pédiatrique, Hôpital Pellegrin-Enfants, CHU de Bordeaux, 33000 Bordeaux, France; Hopital Purpan, 31300 Toulouse, France; Université Paris Saclay, AP-HP, INSERM, Service endocrinologie et diabète de l’enfant, Centre de référence des maladies rares du métabolisme du calcium et du phosphate, Hôpital Bicêtre Paris Saclay, 94270 Le Kremlin-Bicêtre, France; Kyowa Kirin International, Marlow SL7 1HZ, United Kingdom; Kyowa Kirin International, Marlow SL7 1HZ, United Kingdom; Kyowa Kirin International, Marlow SL7 1HZ, United Kingdom; Kyowa Kirin Co. Ltd., Tokyo 194-0023, Japan; Chilli Consultancy Ltd, Salisbury, United Kingdom; College of Architecture, Texas A&M University, College Station, TX 77843, United States; College of Medicine, Texas A&M University, College Station, TX 77807, United States; CHU de Lyon – Hôpital Femme Mère Enfant, 69500 Bron, France

**Keywords:** X-linked hypophosphatemia (XLH), patient-reported outcomes, health-related quality of life, burosumab, registry

## Abstract

X-linked hypophosphatemia (XLH) is a phosphate-wasting disorder caused by increased fibroblast growth factor 23 (FGF23); it leads to skeletal deformities, muscle weakness, and pain. In a pediatric phase 3 trial, the FGF23 inhibitor burosumab improved rickets severity and bone biochemistry. The current study characterizes health-related quality of life (HRQL) in children with XLH using real-world data collected at centers in France for the International XLH Registry from April 2017 to January 2024. Age-appropriate versions of the Pediatric Quality of Life Inventory were completed. Data from the first completion after registry entry were analyzed. Variation in scores by demographic, medical history, and treatment history variables was assessed using bivariate analysis. The data were collected from 96 children (59% female; mean age 8.1 [SD 4.4] yr); 82% were taking burosumab. Mean total, summary, and domain scores were similar in different age groups. Mean total score (74.2 [SD 14.1]), Psychosocial Health Summary (72.0 [16.8]), and Physical Health Summary (78.3 [12.3]) scores were lowest in patients aged 5-7 yr and highest in patients aged 13-17 yr (81.0 [13.3], 79.8 [14.1], and 83.1 [15.2]). The mean Psychosocial Health Summary score was lower than the Physical Health Summary score for all patients combined (77.8 [13.9] vs 81.7 [14.3]). Better total scores were associated with not currently taking phosphate/vitamin D analogs, better Psychosocial Health Summary scores with higher serum phosphate and not taking phosphate/vitamin D analogs, and better Physical Health Summary scores with lower serum PTH and currently taking burosumab. Patients with XLH who were taking burosumab at the time of PedsQL completion had better total and summary scores than children with other chronic musculoskeletal disorders. Children aged 5-7 yr had worse HRQL than a healthy Dutch sample. Overall, better HRQL was associated with higher serum phosphate levels and burosumab treatment.

## Graphical Abstract

**Graphical Abstract:**
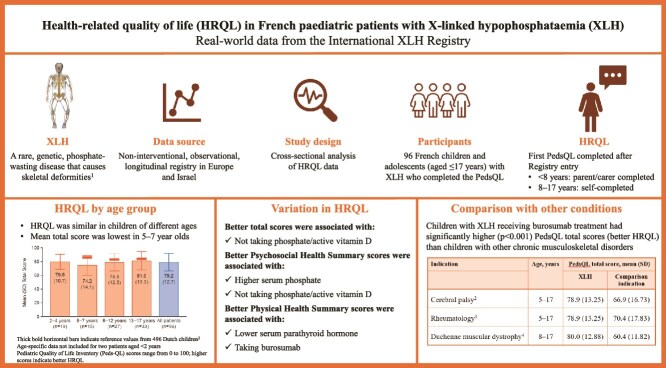

## Introduction

X-linked hypophosphatemia (XLH) is a rare, genetic, phosphate-wasting disorder that is caused by loss-of-function variants in the *PHEX* (phosphate-regulating endopeptidase homologue, X-linked) gene.^1^^,^^2^ This leads to an increase in circulating levels of fibroblast growth factor 23 (FGF23), which causes renal phosphate wasting, reduced production of 1,25(OH)_2_D_3_ and impaired intestinal absorption of phosphate. Chronic hypophosphatemia and dysregulation of bone metabolism during childhood can compromise bone mineralization, leading to skeletal deformities, delayed and disproportionate growth, and short stature, and can cause defects in dental mineralization.^1^^,^^3–6^ Orthopedic surgery may be required to correct skeletal deformities and gait abnormalities. Children may also experience muscle weakness and pain,^7^ obesity,^8^ and craniosynostosis.^9^

Before the approval of burosumab in Europe in 2018, treatment for XLH in children was supplementation with oral phosphate and vitamin D analogs. However, the therapeutic benefit is considered to be insufficient and variable, the treatment is burdensome, because it needs to be administered several times a day, patients experience gastrointestinal side-effects, and adherence to treatment is often poor.^10^ Patients are also at risk of long-term adverse metabolic effects, such as hypercalciuria (further leading to nephrocalcinosis) and secondary hyperparathyroidism.^9^^,^^10^ Burosumab is a fully human monoclonal antibody that binds to and inhibits the activity of excess circulating FGF23.^11^ In a 64-wk phase 3 trial involving children aged 1-12 yr, burosumab treatment improved rickets severity, growth, and markers of bone biochemistry compared with treatment with phosphate supplements and vitamin D analogs.^12^

Health-related quality of life (HRQL) is an intrinsically multidimensional patient-reported outcome; it is defined by the European Medicines Agency as “the patient’s subjective perception of the impact of his disease and its treatment(s) on his daily life, physical, psychological, and social functioning and well-being.”^13^ Improvement of HRQL is a long-term goal in XLH^14^ and experts have called for HRQL to be assessed in children receiving burosumab for the treatment of XLH.^15^ Even though the impact of XLH on HRQL in adults has been well established, evidence relating to HRQL in pediatric XLH patients is still emerging. HRQL benefits have been reported for pediatric subjects receiving treatment with burosumab.^16^^,^^17^

The objectives of this analysis were to describe the HRQL of pediatric XLH patients in France, using real-world registry data, to examine variation in HRQL according to patient, XLH, and treatment characteristics, and to contextualize the findings by comparing HRQL scores with those from healthy children and children with other chronic musculoskeletal conditions.

## Materials and methods

### Study data

The International XLH Registry was established in August 2017 across sites in Europe and Israel as a non-interventional observational real-world data collection program. It is projected to collect data from approximately 1200 patients spanning 10 yr.^18^^,^^19^ The International XLH Registry includes patients of any age who have a clinical presentation or radiological, biochemical, genetic, or family mapping investigation results that support a diagnosis of XLH at the enrollment visit. Patients who have a non-*PHEX* mutation confirmed by genetic test are not eligible. Patients are enrolled regardless of treatment for XLH (oral phosphate and/or vitamin D analogs, burosumab, or currently untreated); however, patients involved in an interventional clinical trial are not eligible until completion of that trial.

The International XLH Registry is administered in accordance with the recommendations guiding physicians in biomedical research involving human subjects that were adopted in 1964 by the 18th World Medical Assembly, Helsinki, Finland, with later revisions. For pediatric patients, parental informed consent for inclusion in the International XLH Registry was obtained from the child’s legally designated representative in line with national guidance. Assent was also sought from children of applicable age in line with national guidance.

### Data collection

The International XLH Registry does not have any predetermined follow-up requirements. Physicians are asked to promptly report accurate and complete data in the Registry electronic data capture system after a patient visit, when new information is available, or at least annually. The registry protocol does not require any additional clinical interventions other than standard clinical practice. Only data collected during standard routine examinations are recorded in the registry. All the data for French pediatric patients were recorded by an individual designated data abstractor. All available retrospective information on general medical history is collected regardless of the time elapsed between registry entry and the XLH diagnosis. Data capture will continue until the end of the XLH International Registry, subject discontinuation, loss to follow-up, or death, whichever occurs first.

Following a protocol amendment in October 2021, pediatric patients were asked to complete the Pediatric Quality of Life Inventory (PedsQL) at clinic visits; completion was recommended but optional. The PedsQL comprises four domains—Physical, Emotional, Social, and School Functioning. It also generates two summary scores—Physical Health and Psychosocial Health—and a total score.^20^ The recall period is 1 mo. Linguistically validated and age-appropriate versions of the PedsQL in French are available for self-completion by children aged 8-17 yr and for completion by parents/guardians for those aged <8 yr.

### Analyses

The current analysis used data through January 2024 from pediatric patients (age <18 yr at registry entry) who enrolled in the International XLH Registry at French centers between April 2017 and October 2023.

Patient characteristics, including demographics and medical history, are summarized by the number of subjects, mean, SD, and range for continuous variables, and the number and percentage of patients for categorical variables. Demographic variables included age, age group, and sex. Medical history variables included height, weight, BMI, age at XLH diagnosis, time from diagnosis to registry entry, serum markers of bone biochemistry (phosphate, alkaline phosphatase [ALP], 1,25(OH)_2_D_3_, and PTH) current treatment, and clinical and surgical histories.

The first PedsQL completion after registry entry was analyzed regardless of the time since registry entry. The PedsQL was scored according to the scoring guide.^21^ All domain, summary, and total scores range from 0 to 100, with higher scores indicating better HRQL/fewer problems or symptoms. Scores are presented for each age-specific version and combined across all age groups. If >50% of the items in a domain were missing, the domain score was not calculated. If ≥50% of the items were completed, the domain score was calculated as the mean of the completed items in the domain. The same rule applied to the total score. If >50% of all items were missing, the total score was not calculated.

Variation in PedsQL scores by demographic, medical history, and treatment history variables was assessed using Mann–Whitney (2 categories), Kruskall–Wallis (≥3 categories), and Spearman’s rank correlation coefficient (ordinal/continuous data).

PedsQL scores for the pediatric patients with XLH who were taking burosumab at the time of PedsQL completion were also compared with scores from pediatric populations with other chronic musculoskeletal conditions—cerebral palsy,^22^ rheumatological conditions,^23^ and Duchenne muscular dystrophy^24^—using Wilcoxon one-sample signed rank tests. The International XLH Registry patients were age-matched to the comparison data.

Statistical significance testing was two-sided at the 5% level. Multiple comparisons were not accounted, because all analyses were performed in an exploratory manner. Analyses were performed using SAS 9.4.

## Results

### Study population

A total of 170 pediatric patients were enrolled in the International XLH Registry at French centers between August 2017 and October 2023, 96 (56%) of whom completed the PedsQL at least once and are included in the current analysis. Demographic and medical history data at registry entry are presented in Table 1. The mean age at registry entry was 8.1 (SD 4.4) yr, and 59% of patients were female. The mean age at XLH diagnosis was 2.1 (2.7) yr. At registry entry, 6.3% of patients had previously undergone corrective orthopedic surgery. Six patients had undergone craniotomy before registry entry. Six had undergone osteotomy and three had also undergone stapling of growth plates. The mean time from registry entry to first PedsQL completion was 2.0 (1.1) yr (range 0.0-4.6). Demographic and medical history characteristics of children who completed the PedsQL were similar to those of the whole pediatric population, other than for age and time since diagnosis (patients who completed the PedsQL were slightly younger [mean ± SD: 8.1 ± 4.4 vs 9.6 ± 5.0 yr] and time since diagnosis was slightly shorter [5.9 ± 4.3 vs 7.3 ± 4.9 yr]).

**Table 1:** Demographic and medical history at International XLH Registry entry for pediatric patients who completed the PedsQL (*n* = 96) and all pediatric patients (*n* = 170).

**Variable**		**PedsQL completers (*n* = 96)**	**All pediatric patients (*n* = 170)**
**Sex, *n* (%)**	Female	57 (59.4)	101 (59.4)
	Male	39 (40.6)	69 (40.6)
**Age (yr), mean ± SD** **Range**		8.1 ± 4.4 0.3 to 16.0	9.6 ± 5.0 0.2 to 17.9
**Height Z-score**	*n* Mean ± SD Range	86 −1.5 ± 1.1 −3.6 to 1.1	150 −1.4 ± 1.3 −11.4 to 1.4
**Weight Z-score^a^**	*n* Mean ± SD Range	61 −0.3 ± 1.1 −2.4 to 5.0	90 −0.1 ± 1.2 −2.4 to 5.0
**BMI (kg/m^2^) Z-score**	*n* Mean ± SD Range	86 0.8 ± 1.4 −1.9 to 9.1	148 1.0 ± 1.9 −2.1 to 17.5
**Age at diagnosis (yr)**	*n* Mean ± SD Range	94 2.1 ± 2.7 0.0 to 14.9	165 2.1 ± 2.5 0.0 to 14.9
**Time since diagnosis (yr)**	*n* Mean ± SD Range	94 5.9 ± 4.3 0.0 to 13.9	165 7.3 ± 4.9 0.0 to 17.4
**Reported clinical history, *n* (%)** ** Nephrocalcinosis** ** Tooth abscess** ** Excessive cavities** ** Genu varum** ** Genu valgum** ** Intoeing** ** Tibial torsion** ** Club foot deformity** ** Windswept deformity** ** Craniosynostosis**		37 (38.5) 35 (36.5) 22 (22.9) 43 (44.8) 24 (25.0) 3 (3.1) 0 (0) 0 (0) 0 (0) 28 (29.2)	65 (38.2) 62 (36.5) 36 (21.2) 79 (46.5) 42 (24.7) 8 (4.7) 1 (0.6) 1 (0.6) 0 (0) 46 (27.1)
**Procedure, *n* (%)**			
** Craniotomy**		6 (6.3)	13 (7.6)
**Corrective surgery**^b^		6 (6.3)	18 (10.6)
** Leg lengthening**		0	0
** Osteotomy**		6 (6.3)	14 (8.2)
** Stapling of growth plates**		3 (3.1)	8 (4.7)
** Tibial torsion**		0	0
**Number of corrective surgeries**	0	90 (93.8)	152 (89.4)
	1	3 (3.1)	12 (7.1)
	2	2 (2.1)	4 (2.4)
	3	1 (1.0)	2 (1.2)

a *n* = 61/90, World Health Organization weight-for-age reference data are not available beyond age 10 yr.

b Patients may have undergone more than one corrective surgery. Corrective surgeries are: leg lengthening, osteotomy, stapling of growth plates, and tibial torsion.

Z-scores calculated using World Health Organization reference data.^42^ A positive Z-score shows that the value lies above the mean; a negative Z-score shows that the value lies below the mean, a Z-score of 1.0 indicates that a value that is 1 SD from the mean.

Abbreviations: PedsQL, Pediatric Quality of Life Inventory; XLH, X-linked hypophosphatemia.

Treatment at registry entry and PedsQL completion is reported in Table 2: 89.6% of patients were receiving treatment for XLH at registry entry, increasing to 95.8% at PedsQL completion. At PedsQL completion, most patients were receiving treatment with burosumab (82.3%); 13.5% were taking phosphate supplements and/or vitamin D analogs. About 21% of patients were receiving growth hormone treatment at registry entry and PedsQL completion.

**Table 2:** Treatment for XLH at International XLH Registry entry and first PedsQL completion (*n* = 96).

		**At registry entry** ^**a**^	**At PedsQL completion** ^**a**^
**Currently receiving treatment for XLH, *n* (%)**		86 (89.6)	92 (95.8)
**Current treatment, *n* (%)** ** Burosumab** ** Phosphate and/or vitamin D analogs **		51 (53.1) 36 (37.5)	79 (82.3) 13 (13.5)
**Currently receiving growth hormone, *n* (%)**		20 (20.8)	20 (20.8)
**Time on burosumab (mo)^b^**	*n*	51	79
	Mean ± SD	18.8 ± 10.4	34.2 ± 20.8
	Range	0.1 to 59.7	0.2 to 93.4
**Time on phosphate and/or vitamin D analogs (mo)^b^**	*n*	36	13
	Mean ± SD	61.5 ± 59.7	92.4 ± 78.9
	Range	0.9 to 228.7	9.6 to 263.6
**Age initiating burosumab (yr)^b^**	*n*	51	79
	Mean ± SD	7.5 ± 3.7	7.6 ± 4.4
	Range	0.6 to 14.5	0.6 to 17.4
**Age initiating growth hormone (yr)^b^**	*n*	21	20
	Mean ± SD	7.9 ± 3.1	8.1 ± 3.1
	Range	2.7 to 12.7	2.7 to 12.7

a On day of registry entry or PedsQL completion, a visit window was not applied.

b Patients receiving treatment at International XLH Registry entry/PedsQL completion.

Abbreviations: PedsQL, Pediatric Quality of Life Inventory; SD, standard deviation; XLH, X-linked hypophosphatemia.

Biochemistry values at registry entry and at the time of PedsQL completion are presented in Table S1. Serum concentrations for phosphate, ALP, 1,25(OH)_2_D_3_, and PTH at first PedsQL completion were similar to concentrations at registry entry, and similar proportions of patients had values within the normal range other than for serum phosphate.

### HRQL burden at first PedsQL completion

**Figure 1:**
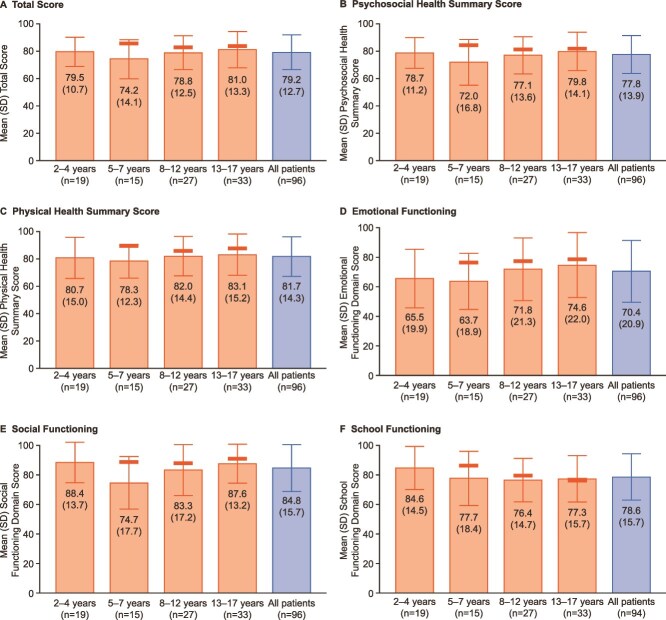
PedsQL domain, summary, and total scores (*n* = 96). PedsQL scores range from 0 to 100, with higher scores indicating better health-related quality of life. Thick bold horizontal bars indicate reference values from 496 Dutch children (not available for children aged <5 yr, or all ages combined).^36^ Age-specific data not included for two patients aged <2 yr. Results for the Physical Functioning domain are not shown because this domain is the same as the Physical Health Summary score. Abbreviation: PedsQL, Pediatric quality of life inventory.

PedsQL total, summary, and domain scores were similar for the different age groups, as shown in Figure 1. Mean total scores (74.2 [14.1]), Psychosocial Health Summary (72.0 [SD 16.8]), and Physical Health Summary (78.3 [12.3]) scores were lowest in patients aged 5-7 yr and highest in patients aged 13-17 yr (81.0 [13.3], 79.8 [14.1], and 83.1 [15.2], respectively) (Figure 1). For patients of all ages combined, the Psychosocial Health Summary score (77.8 [13.9]) was lower than the Physical Health Summary score (81.7 [14.3]). Mean domain scores were lowest (worse HRQL) in 5-7-yr-olds for two of the three domains: Emotional Functioning (63.7 [18.9]) and Social Functioning (74.7 [17.7]). Scores for Emotional Functioning were highest (better HRQL) in patients aged 13-17 yr (74.6 [22.0]). Scores for Social Functioning (88.4 [13.7]) and School Functioning (84.6 [14.5]) were highest in patients aged 2-4 yr. For patients of all ages combined, domain scores were highest (better HRQL) for Social Functioning (84.8 [15.7]) and lowest (worse HRQL) for Emotional Functioning (70.4 [20.9]).

**Figure 2:**
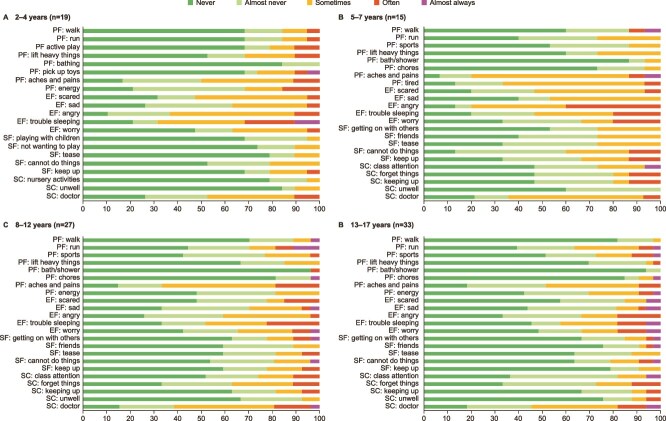
PedsQL item responses at first completion. Abbreviations: PedsQL, Pediatric quality of life inventory; PF, physical functioning; EF, emotional functioning; SF, social functioning.

Figure 2 presents the responses to each PedsQL item by age group. For patients aged 2-4 yr, the greatest burden (based on proportions of patients reporting “often” or “almost always”) was reported for “trouble sleeping” (31.6%), followed by “low energy level” (15.8%). For patients aged 5-7 yr, the greatest burden was reported for “feeling angry” (40.0%), followed by “trouble sleeping” (20.0%) and “worrying about what will happen to him or her” (20.0%). For patients aged 8-12 yr, the greatest burden was reported for “I have trouble sleeping” (22.2%), followed by “I miss school to go to the doctor or hospital” (19.2%). For patients aged 13-17 yr, the greatest burden was for “I feel sad or blue,” “I feel angry,” “I worry about what will happen to me,” and “I miss school to go to the doctor or hospital” (all 18.2%).

### Variation in HRQL by demographic, medical history, and treatment characteristics

The evaluation of variation in HRQL by demographics, medical history, treatment history, and current treatment is presented in Tables 3 and 4. Variation in HRQL by biochemistry is presented in Table S2 and variation in HRQL by clinical characteristics (all non-significant) in Table S3.

**Table 3:** Variation in PedsQL total, summary, and domain scores according to demographic and medical history variables.

**Characteristic**		***n***	**PedsQL total score**		**Psychosocial Health Summary**		**Physical Health Summary**		**Emotional Functioning**		**Social Functioning**		**School Functioning**	
			**Mean or corr. coeff.** ^a^	***p*-value**	**Mean or corr. coeff.** ^a^	***p*-value**	**Mean or corr. coeff.** ^a^	***p*-value**	**Mean or corr. coeff.** ^a^	***p*-value**	**Mean or corr. coeff.** ^a^	***p*-value**	**Mean or corr. coeff.** ^a^	***p*-value**
**Age (yr)**		96	0.126	.220	0.111	.283	0.112	.278	0.245	**.016**	0.039	.707	−0.147	.157
**Sex**	Female	57	78.6	.578	77.5	.612	80.8	.683	71.4	.574	84.0	.228	77.4	.305
	Male	39	79.9		78.1		83.0		69.0		85.9		80.3	
**Height Z-score^b^**		86	0.071	.515	0.048	.663	0.102	.351	0.071	.519	−0.022	.841	0.047	.667
**Weight Z-score^b^**		61	−0.025	.848	−0.064	.625	0.025	.848	0.020	.881	−0.104	.426	−0.094	.479
**BMI Z-score^b^**		86	−0.100	.359	−0.073	.506	−0.145	.184	−0.079	.467	−0.087	.425	0.006	.955
**Age at diagnosis**		94	−0.103	.325	−0.119	.254	−0.079	.451	−0.070	.503	−0.152	.143	−0.096	.365
**Time since diagnosis**		94	0.124	.232	0.096	.356	0.166	.111	0.165	.112	0.095	.362	−0.121	.250
**Time from enrollment to PedsQL completion**		96	0.120	.243	0.094	.363	0.140	.174	0.185	.071	0.044	.672	−0.085	.414

There are no results for the Physical Functioning domain because it is identical to the Physical Health Summary.

a Values are mean PedQL scores for categorical data (eg, male/female), which were compared using the Mann–Whitney test. Spearman’s rank correlation coefficient is reported for ordinal/continuous variables. Significant results (*p* < .05) are shown in bold.

b ±1 yr of enrollment.

Abbreviations: corr. coeff., correlation coefficient; PedsQL, Pediatric Quality of Life Inventory.

**Table 4:** Variation in PedsQL total, summary, and domain scores by treatment history and current treatment variables.

**Characteristic**		***n***	**PedsQL total score**		**Psychosocial Health Summary**		**Physical Health Summary**		**Emotional Functioning**		**Social Functioning**		**School Functioning**	
			**Mean or corr. coeff.** ^a^	***p*-value**	**Mean or corr. coeff.** ^a^	***p*-value**	**Mean or corr. coeff.** ^a^	***p*-value**	**Mean o corr. coeff.** ^a^	***p*-value**	**Mean or corr. coeff.** ^a^	***p*-value**	**Mean or corr. coeff.** ^a^	***p*-value**
**Burosumab naïve**	Yes	15	73.7	.053	72.2	.060	76.0	.078	66.7	.530	75.3	**.029**	73.2	.257
	No	81	80.2		78.8		82.7		71.1		86.5		79.4	
**Current treatment with** **burosumab**	Yes	79	80.1	.075	78.6	.154	82.9	**.049**	70.8	.773	86.4	.052	79.3	.390
	No	17	74.7		73.9		75.7		68.6		77.4		74.8	
**Time on burosumab (mo)**		79	0.040	.725	0.053	.644	−0.014	.901	0.097	.396	0.039	.734	−0.101	.377
**Age started burosumab**		79	0.183	.106	0.170	.135	0.148	.194	0.300	**.007**	0.100	.381	−0.186	.101
**Current treatment with phosphate and/or vitamin D analogs**	Yes	13	73.0	**.038**	71.4	**.047**	75.5	.055	71.2	.475	75.0	**.025**	73.5	.360
	No	83	80.1		78.7		82.6		65.4		86.3		79.3	
**Time on phosphate and/or vitamin D analogs**		13	−0.330	.271	−0.433	.140	0.070	.821	−0.226	.457	−0.423	.150	−0.095	.770
**Growth hormone naïve**	Yes	72	78.9	.685	77.6	.822	81.2	.753	70.3	.809	83.5	.182	79.7	.224
	No	24	79.8		78.1		83.0		70.8		88.5		75.3	
**Current treatment with growth hormone**	Yes	20	78.6	.903	76.9	.836	81.6	.831	70.2	.885	87.5	.369	73.4	.094
	No	76	79.3		78.0		81.7		70.5		84.1		80.0	
**Age initiating growth hormone (yr)**		21	−0.124	.592	−0.141	.541	−0.217	.346	−0.223	.331	−0.146	.527	−0.050	.828

a Values are mean PedsQL scores for categorical data (eg, male/female), which were compared using the Mann–Whitney test. Spearman’s rank correlation coefficient is reported for ordinal/continuous variables.

Note that each treatment was a distinct dichotomous variable; patients may have been receiving more than one treatment.

Significant results (*p* < .05) are shown in bold.

There are no results for the Physical Functioning domain because it is identical to the Physical Health Summary.

Abbreviations: corr. coeff., correlation coefficient; PedsQL, Pediatric Quality of Life Inventory.

Better total scores were associated with not currently receiving treatment with phosphate and/or vitamin D analogs (*p* = .038). Better Psychosocial Health Summary scores were associated with higher serum phosphate levels (*p* = .038) and not currently receiving treatment with phosphate and/or vitamin D analogs (*p* = .047). Better Physical Health Summary scores were associated with lower serum PTH levels (*p* = .039) and currently receiving treatment with burosumab (*p* = .049).

At the domain level, better Emotional Functioning was associated with being older (*p* = .016) and starting burosumab at a later age (*p* = .007). Better Social Functioning was associated with prior exposure to burosumab (*p* = .029) and not currently receiving treatment with phosphate and/or vitamin D analogs (*p* = .025). School Functioning scores did not have a significant relationship with any of the variables tested. Results are not reported separately for the Physical Functioning domain, because this is identical to the Physical Health Summary score.

### Comparison of PedsQL scores in XLH with other indications in pediatric patients

Table 5 compares PedsQL scores from the current International XLH Registry sample with published PedsQL scores for other chronic musculoskeletal conditions (cerebral palsy, rheumatology, and Duchenne muscular dystrophy) and a healthy sample of Dutch children. Patients with XLH had significantly better HRQL than the comparison indications on the total score, Psychosocial Summary Score, Physical Health Summary score, and School Functioning domain, and for most comparisons for the Emotional Functioning domain except for cerebral palsy, rheumatology, and Duchenne muscular dystrophy self-reports, and for rheumatology self-report in the Social Functioning domain. Patients aged 8-12 and 13-17 yr with XLH did not have significantly different HRQL from a healthy Dutch sample on the total, summary, or any domain scores. Patients aged 5-7 yr with XLH had significantly worse HRQL than the healthy Dutch sample on total, summary, and all domain scores, except for the School domain, where there was no significant difference.

**Table 5:** Comparison of PedsQL total, summary, and domains scores in pediatric patients receiving burosumab in the current study, pediatric populations with other chronic musculoskeletal conditions and a healthy sample of children.

**Indication and source**	**Age, yr**	**Report**	***n***	**Total score**	**Summary scores**		**Domain scores** ^a^		
					**PSS**	**PHS**	**Emotional**	**Social**	**School**
**XLH** **(current analysis)**	5-7	Proxy	15	74.2 (14.11)	72.0 (16.76)	78.3 (12.30)	63.7 (18.94)	74.7 (17.67)	77.7 (18.41)
	8-12	Self	27	78.8 (12.45)	77.1 (13.57)	82.0 (14.38)	71.8 (21.28)	83.3 (17.15)	76.4 (14.69)
	13-17	Self	33	81.0 (13.34)	79.8 (14.11)	83.1 (15.15)	74.6 (21.99)	87.6 (13.18)	77.3 (15.72)
	5-17	Proxy/Self	75	78.9 (13.25)	77.3 (14.58)	81.8 (14.27)	71.4 (21.28)	83.5 (16.15)	77.1 (15.71)
	8-17	Self	60	80.0 (12.88)	78.6 (13.82)	82.6 (14.69)	73.4 (21.54)	85.7 (15.11)	76.9 (15.14)
**Cerebral palsy^22^**	5-18	Self	77	66.9 (16.73) ***p* < .001**	68.1 (16.52) ***p* < .001**	64.4 (22.08) ***p* < .001**	68.6 (22.93) *p* = 0.097	70.5 (19.26) ***p* < .001**	65.6 (22.18) ***p* < .001**
		Proxy	224	51.3 (18.00) ***p* < .001**	56.0 (16.98) ***p* < .001**	43.2 (27.59) ***p* < .001**	62.7 (19.55) ***p* < .001**	52.1 (21.97) ***p* < .001**	52.0 (21.41) ***p* < .001**
**Rheumatology^b^^23^**	5-18	Self	336	70.4 (17.83) ***p* < .001**	72.7 (17.07) ***p* = .004**	66.0 (23.81) ***p* < .001**	68.3 (22.85) *p* = .097	80.1 (19.10) *p* = .154	69.9 (19.97) ***p* < .001**
		Proxy	357	68.7 (19.32) ***p* < .001**	71.3 (18.49) ***p* < .001**	64.1 (25.01) ***p* < .001**	66.3 (22.27) ***p* = .011**	76.5 (20.56) ***p* < .001**	70.6 (22.43 ***p* = .001**
**Duchenne muscular dystrophy^24^**	8-18	Self	43	60.4 (11.82) ***p* < .001**	69.0 (12.33) ***p* < .001**	44.1 (21.83) ***p* < .001**	70.3 (15.94) *p* = 0.123	73.0 (15.97) ***p* < .001**	64.0 (15.16) ***p* < .001**
**Dutch healthy sample^36^**	5-7	Self	76	85.3 (8.59) ***p* = .007**	83.5 (9.70) ***p* = .014**	88.7 (9.13) ***p* = .005**	76.4 (13.80) ***p* = .024**	87.8 (11.70) ***p* = .017**	86.5 (10.76) *p* = .164
	8-12	Self	192	82.3 (8.83) *p* = .175	80.8 (10.34) *p* = .183	85.3 (8.85) *p* = .606	76.9 (13.76) *p* = .466	86.5 (12.24) *p* = .408	76.9 (13.76) *p* = .725
	13-18	Self	148	83.1 (8.99) *p* = .827	81.2 (10.22) *p* = .965	86.8 (9.21) *p* = .455	77.5 (15.01) *p* = .773	90.1 (11.37) *p* = .476	75.9 (12.68) *p* = .488

Scores are mean (SD); *p*-value is the comparison with the published score with the corresponding XLH age-matched population. Significant differences (p<0.05) are shown in bold.

PedsQL scores for International XLH Registry patients were compared with PedsQL scores from disease-specific samples using Wilcoxon one-sample signed rank tests. Samples were age matched; n = 75 XLH pediatric patients aged 5-17 yr, *n* = 60 aged 8-17 yr.

All domain, summary, and total scores range from 0 to 100, with higher scores indicating better HRQL/fewer problems or symptoms.

All comparison studies were in the US.

Abbreviations: PedsQL, Pediatric Quality of Life Inventory; PHS, Physical Health Summary; PSS, psychosocial summary; XLH, X-linked hypophosphatemia.

a The Physical Function domain score is the same as the Physical Health Summary score so the results are not shown.

b Juvenile arthritis, fibromyalgia, spondyloarthritis, systemic lupus erythematosus.

## Discussion

This analysis explores the HRQL burden in a large cohort of French pediatric patients with XLH using real-world data. It has also assessed variation in HRQL according to patient, XLH, and treatment characteristics, showing that better overall HRQL was associated with not currently receiving treatment with phosphate supplements and/or vitamin D analogs, better Psychosocial Health Summary scores were associated with higher serum phosphate levels and not currently receiving treatment with phosphate supplements and/or vitamin D analogs, and better Physical Health Summary scores were associated with currently receiving treatment with burosumab and lower serum PTH levels. The results also contextualize the HRQL burden in those taking burosumab vs children with other chronic musculoskeletal conditions and a healthy population. The International XLH Registry cohort had significantly better HRQL than children with cerebral palsy, rheumatological conditions, or Duchenne muscular dystrophy and did not have significantly different HRQL scores from a healthy sample, with the exception of 5-7-yr-olds with XLH, who had worse HRQL than their healthy peers (Table 5).

At PedsQL completion, 82% of children were taking burosumab and 14% were taking phosphate supplements and/or vitamin D analogs; 4% were taking neither treatment. Children taking burosumab had numerically better overall HRQL and Psychosocial Health Summary scores than those not taking it (a mixed group comprising the 14% [*n* = 13] taking phosphate supplements and/or vitamin D analogs and the 4%[(*n* = 4] taking neither of these treatments). Physical Health Summary scores were significantly higher in those currently receiving burosumab treatment than in the mixed treatment group. The therapeutic benefit of phosphate and/or vitamin D is considered to be insufficient and variable, the treatment is burdensome, patients experience gastrointestinal side-effects, and are at risk of long-term adverse metabolic effects.^9^^,^^10^ However, this analysis is confounded, because those taking burosumab are compared with a mixed group of those taking phosphate supplements and/or vitamin D or neither treatment; this needs to be taken into consideration when designing future analyses. In addition, this is a cross-sectional analysis so causality cannot be determined.

Burosumab has been available in France for the second-line treatment of XLH in children since 2018 and for all children since January 2024. The uptake of burosumab as the primary treatment for pediatric XLH in France is apparent from the current data: at registry entry, 53% of patients were taking burosumab, increasing to 82% by first PedsQL completion, when patients had been receiving burosumab for an average of 2 yr and 10 mo, and some had been receiving it for more than 7 yr. By contrast, only 13.5% of patients were taking phosphate supplements and/or vitamin D analogs. Clinical trial data have shown improvements in HRQL in pediatric patients treated with burosumab,^12^^,^^16^ and it is therefore likely that burosumab will have a positive effect on PedsQL scores within the International XLH Registry. The mean age at initiating burosumab treatment was 7.6 yr (range 0.6-17.4 yr), but this will decrease in subsequent analyses now that burosumab is available to children of all ages, enabling earlier initiation.

Growth hormone treatment may be used in children with XLH to improve final height.^25^ At the time of PedsQL completion, 21% of patients were taking growth hormone. The current relationship did not identify any relationship between growth hormone treatment and HRQL in the current analysis; however, any possible relationship between the use of growth hormone and HRQL may become apparent when final height is reached.

In this study, HRQL in patients younger than 8 yr was proxy-reported by parents/caregivers, whereas older children self-reported using age-appropriate versions of the PedsQL. This approach has the advantage of self-reported HRQL data from the older children but also ensures that the HRQL of younger children, who may not be able to reliably self-report, is incorporated into the analysis. A large study has demonstrated the PedsQL to be a reliable and valid measure for parent-proxy report; although the authors advocate for pediatric self-report, there remains a fundamental role for parent proxy-report in pediatric studies.^26^ Nevertheless, it is important to consider that these different reporting formats may be a confounder. It may explain why, for example, patients aged 5-7 yr had the worst scores on PedsQL total, summary, and all domain scores. However, published evidence comparing child self-reporting with parent-proxy reporting of HRQL shows no consistent pattern: studies describe both under and overreporting of HRQL by parents compared with children self-reporting, and some studies report a mixed-pattern across HRQL domains.^27–35^ In the current study, children aged 2-4 yr did not have the same consistently lower HRQL than the older age groups, even though their HRQL was proxy-reported by parents/caregivers. The PedsQL data for healthy Dutch children^36^ were based on the same methodology—caregiver reported for children under 8 yr of age and self-reported for older children. The finding in the current study may be because children in this age group have not yet had time to come to terms with their diagnosis, whereas older children are increasingly affected by the musculoskeletal effects of XLH (eg, joint pain, mobility, and short stature), dental effects, and burden of treatment; they may also be starting to undergo corrective surgery. They are also attending school and mixing with healthy peers, possibly making them more aware of their limitations and increasing the emotional impact of the XLH diagnosis.^9^^,^^37^^,^^38^

Analysis of the PedsQL item-level data is particularly informative, both overall and with regard to the lower HRQL in children aged 5-7 yr. HRQL burden is influenced by sleep issues for all age groups except those aged 13-17 yr. Emotional concerns dominate from age 5 yr onwards, and older age groups are missing school to attend doctor and hospital appointments. The greater impact of anger in those aged 5-7 yr compared with the younger age group is striking, alongside notable issues related to worry. This supports the proposal that this age group may be coming to terms with their diagnosis. The older age groups show more frequent reporting of issues being apparent “almost always” compared with younger age groups, reflecting the progressive nature of XLH, although this is balanced by greater reporting for most items of “never” or “almost never” having issues.

This study provides the first real-world evidence of a relationship between HRQL and bone biochemistry markers in pediatric XLH: better Psychosocial Health Summary scores were significantly associated with higher serum phosphate concentrations. The improvement in serum phosphate concentrations in children with XLH (up to 12 yr) has been established in the clinical trial setting^12^^,^^39^^,^^40^ and was associated with improvements in patient-reported pain, physical function mobility, fatigue, and HRQL.^16^ Treatment with burosumab to correct serum phosphate levels to the normal range could be experienced by patients through improved HRQL. This is supported by the finding in the current study that better Physical Health Summary scores were associated with current burosumab treatment, and better overall HRQL and Psychosocial Summary scores were associated with not currently receiving treatment with phosphate supplements and/or vitamin D analogs.

The finding that Emotional Functioning was related to both older age and starting burosumab at a later age may arise because these two items capture similar information: older patients started burosumab at an older age because this treatment has only recently become available for use in France. This needs to be explored through assessment of multicollinearity and multivariable analysis in a larger sample. School Functioning was not significantly related to any of the variables assessed in this analysis. A future analysis could explore the relationship between School Functioning and healthcare utilization as it is noted that, at the item level, all age groups reported burden in terms of missing school/daycare to go to the doctor or hospital.

Patients had stable bone biochemistry markers (serum phosphate, ALP, 1,25(OH)_2_D_3_, and PTH) from registry entry through to first PedsQL completion, a mean period of 2 yr (range 0-4.6 yr). Mean serum phosphate remained stable across these two time points. It is important to note that the classification of subjects as having “normal” or “abnormal” biochemistry was made at the site level based on local reference ranges. In addition, substantial biochemistry data at PedsQL completion (±1 yr) were missing (eg, 44% with serum phosphate data at PedsQL completion).

This study has several limitations. This is a cross-sectional analysis of the first PedsQL completed in the International XLH Registry; cross-sectional research cannot establish cause-effect relationships or change over time and may be susceptible to recall bias,^41^ although recall bias was not an issue in this study, because medical history data were captured retrospectively from patient records. The cross-sectional analysis identifies potential relationships that can be evaluated in longitudinal analysis to determine the direction of causality, as would be the case when considering the impact of continued burosumab treatment on HRQL in the real-world setting, for example. As registry data accumulate, the impact of burosumab in patients who started treatment at a young age can be explored and compared with patients who started treatment later in the disease course; the current data include patients who started burosumab treatment as late as 17 yr of age, with a mean age at treatment initiation of 7.6 yr. The timing of PedsQL completion, and particularly the interval after study enrollment, varied in the current study because the PedsQL was added to the study protocol after the study started. The timing of PedsQL completion relative to treatment administration also varied given the real-world setting. The mean time from registry entry to first PedsQL completion was 2.0 yr, which means that some of the demographic and medical history variables that were compared with PedsQL scores may have changed by the time of PedsQL completion (eg, height, weight, BMI Z-scores, time since diagnosis, number of corrective surgeries, and clinical history). The univariate analysis involved testing multiple variables of interest without correction, which increases the likelihood of a type 1 error. The results reported here should therefore be considered exploratory, and contributing to hypothesis development for a future multi-country analysis that will include evaluation of variation in HRQL using multivariable models. This is a single country study and its generalizability beyond France may be limited. PedsQL data are also being captured via the International XLH Registry in Italy, Spain, Sweden, and the UK. An analysis including data from these countries is planned. The HRQL data reported here comprise a mix of caregiver- and self-report. Patient self-report is considered the gold standard but this is not feasible in younger children. Healthcare utilization data were not considered in this analysis; however, this may have a relationship with HRQL and warrants investigation.

In this analysis of real-world data from 96 French pediatric patients with XLH, 82% of whom were receiving burosumab treatment at the time of PedsQL completion, HRQL detriment was apparent compared with a healthy sample; however, HRQL scores in those taking burosumab were significantly higher than in children with other chronic musculoskeletal conditions. Children aged 5-7 yr had the worst HRQL in the current analysis. Interestingly, children with higher serum phosphate levels and those receiving burosumab treatment had better HRQL scores.

## Ethics approval statement

The International XLH Registry is administered in accordance with the recommendations guiding physicians in biomedical research involving human subjects that were adopted in 1964 by the 18th World Medical Assembly, in Helsinki, Finland, with later revisions.

## Patient consent statement

For pediatric patients, parental informed consent for inclusion in the International XLH Registry was obtained from the child’s legally designated representative in line with national guidance. Assent was also sought from children of applicable age in line with national guidance.

## Supplementary Material

XLH_Registry_PedsQL_manuscript_supplementary_resubmission_09JUN25_CLEAN_ziaf142

## Data Availability

Data that underlie the results reported in this article may be requested. Kyowa Kirin International will review requests individually to determine whether requests are legitimate, relevant, and meet sound scientific principles and are within the scope of the participants’ informed consent.
