# Correction to: Health-related quality of life in French pediatric patients with X-linked hypophosphatemia: real-world data from the International XLH Registry

**DOI:** 10.1093/jbmrpl/ziag133

**Published:** 2026-08-17

This is a correction to: Agnès Linglart, Cyril Amouroux, Iva Gueorguieva, Jerome Harambat, Jean-Pierre Salles, Diana-Alexandra Ertl, Kerry Sandilands, Angela Rylands, Angela Williams, Haruka Ishii, Annabel Bowden, James W Varni, Justine Bacchetta, Health-related quality of life in French pediatric patients with X-linked hypophosphatemia: real-world data from the International XLH Registry, *JBMR Plus*, Volume 9, Issue 10, October 2025, ziaf142, https://doi.org/10.1093/jbmrpl/ziaf142

In the originally published version of the paper, an older version of the supplementary data file was published. The correct file is now published with the paper instead of that attached to this notice.

