# Supplementary material for: Health-related quality of life in French pediatric patients with X-linked hypophosphatemia: real-world data from the International XLH Registry
Source: JBMR Plus. 2025 Aug 30;9(10):ziaf142. doi: 10.1093/jbmrpl/ziaf142 (PMC12478027; doi:10.1093/jbmrpl/ziaf142)
Supplement: XLH_Registry_PedsQL_manuscript_supplementary_25JAN25_submission_ziaf142 [file xlh_registry_pedsql_manuscript_supplementary_25jan25_submission_ziaf142.docx]

**Supplementary Materials**

**Supplementary Table 1: Biochemistry at Registry entry and first PedsQL completion (n=96)**

|  |  | **At Registry entry**  **(±1 year^a^)** | **At PedsQL completion**  **(±1 year^b^)** |
| --- | --- | --- | --- |
| Serum phosphate (mmol/L) | n  Mean ± SD  Range  Abnormal, n (%)  Normal, n (%) | 93  1.01 ± 0.21  0.48 to 1.44  51 (54.8)  42 (45.2) | 42  0.98 ± 0.22  0.60 to 1.43  30 (71.4)  12 (28.6) |
| Serum ALP (u/L) | n  Mean ± SD  Range  Abnormal, n (%)  Normal, n (%) | 93  390 ± 166  133 to 1101  49 (52.7)  44 (47.3) | 42  371 ± 185  81 to 1031  17 (40.5)  25 (59.5) |
| Serum 1,25 dihydroxy vitamin D (pg/mL) | n  Mean ± SD  Range  Abnormal, n (%)  Normal, n (%) | 63  68 ± 25  8 to 166  27 (42.9)  36 (57.1) | 28  67 ± 17  27 to 112  10 (35.7)  18 (64.3) |
| Serum PTH (pmol/L) | n  Mean ± SD  Range  Abnormal, n (%)  Normal, n (%) | 88  50 ± 29  5 to 208  13 (14.8)  75 (85.2) | 37  55 ± 28  15 to 123  5 (13.5)  32 (86.5) |
| ^a^+/- 1 year enrolment  ^b^+/- 1 year PedsQL completion  ALP, alkaline phosphatase; PedsQL, Pediatric Quality of Life Inventory; PTH, parathyroid hormone; SD, standard deviation  Abnormal/normal was determined at the site level based on local reference ranges. | | | |

**Supplementary Table S2 Clinical and surgical history**

| **Characteristic** | | **n** | **Total score** | | **Psychosocial Health Summary** | | **Physical Health Summary** | | **Emotional Functioning** | | **Social Functioning** | | **School Functioning** | |
| --- | --- | --- | --- | --- | --- | --- | --- | --- | --- | --- | --- | --- | --- | --- |
|  |  |  | **Mean or corr. coeff.** | **p value** | **Mean or corr. coeff.** | **p value** | **Mean or corr. coeff.** | **p value** | **Mean or corr. coeff.** | **p value** | **Mean or corr. coeff.** | **p value** | **Mean or corr. coeff.** | **p value** |
| Nephrocalcinosis | No | 59 | 79.1 | 0.895 | 77.5 | 0.783 | 82.0 | 0.800 | 68.9 | 0.302 | 85.3 | 0.845 | 79.0 | 0.611 |
|  | Yes | 37 | 79.3 |  | 78.2 |  | 81.2 |  | 72.8 |  | 84.1 |  | 77.9 |  |
| Tooth abscess | No | 61 | 79.2 | 0.909 | 77.7 | 0.807 | 81.7 | 0.939 | 70.8 | 0.852 | 84.4 | 0.642 | 78.8 | 0.686 |
|  | Yes | 35 | 79.1 |  | 77.8 |  | 81.5 |  | 69.8 |  | 85.4 |  | 78.2 |  |
| Excessive cavities | No | 74 | 80.2 | 0.234 | 78.9 | 0.197 | 82.6 | 0.492 | 71.7 | 0.367 | 85.7 | 0.336 | 80.0 | 0.162 |
|  | Yes | 22 | 75.5 |  | 73.9 |  | 78.6 |  | 66.0 |  | 81.8 |  | 73.9 |  |
| Genu varum | No | 53 | 81.1 | 0.246 | 80.2 | 0.081 | 82.5 | 0.546 | 72.8 | 0.169 | 86.9 | 0.175 | 81.6 | 0.127 |
|  | Yes | 43 | 76.8 |  | 74.7 |  | 80.7 |  | 67.5 |  | 82.2 |  | 75.0 |  |
| Genu valgum | No | 72 | 78.7 | 0.672 | 77.7 | 0.889 | 80.4 | 0.101 | 68.8 | 0.233 | 85.5 | 0.275 | 79.5 | 0.271 |
|  | Yes | 24 | 80.6 |  | 78.0 |  | 85.4 |  | 75.3 |  | 82.7 |  | 76.0 |  |
| Craniosynostosis | No | 68 | 79.7 | 0.617 | 78.3 | 0.540 | 82.2 | 0.818 | 72.6 | 0.125 | 84.5 | 0.444 | 78.1 | 0.504 |
|  | Yes | 28 | 77.8 |  | 76.4 |  | 80.3 |  | 65.1 |  | 85.5 |  | 79.8 |  |
| Number of corrective surgeries | | 96 | −0.089 | 0.390 | −0.086 | 0.406 | −0.101 | 0.328 | 0.002 | 0.981 | −0.163 | 0.112 | −0.136 | 0.192 |
| Insufficient number of patients in each group to undertake the analysis for intoeing, tibial torsion, club foot deformity and windswept deformity.  Variation in PedsQL scores was assessed using Matt–Whitney (2 categories), Kruskall–Wallis (≥3 categories) and Spearman’s rank correlation coefficient (ordinal/continuous data).  None of the results are significant (p<0.05).  There are no results for the Physical Functioning domain because it is identical to the Physical Health Summary. | | | | | | | | | | | | | | |
