# Supplementary material for: Health-related quality of life in French pediatric patients with X-linked hypophosphatemia: real-world data from the International XLH Registry
Source: JBMR Plus. 2025 Aug 30;9(10):ziaf142. doi: 10.1093/jbmrpl/ziaf142 (PMC12478027; doi:10.1093/jbmrpl/ziaf142)
Supplement: Supplementary file 2 [file xlh_registry_pedsql_manuscript_supplementary_resubmission_09jun25_clean_ziaf142.docx]

**Supplementary Materials**

**Supplementary Table S1: Biochemistry at Registry entry and first PedsQL completion (n=96)**

|  |  | **At Registry entry** **(±1 year)** | **At PedsQL completion** **(±1 year)** |
| --- | --- | --- | --- |
| Serum phosphate (mmol/L) | n Mean ± SD Range Abnormal, n (%) Normal, n (%) | 93 1.01 ± 0.21 0.48 to 1.44 51 (54.8) 42 (45.2) | 42 0.98 ± 0.22 0.60 to 1.43 30 (71.4) 12 (28.6) |
| Serum ALP (IU/L) | n Mean ± SD Range Abnormal, n (%) Normal, n (%) | 93 390 ± 166 133 to 1101 49 (52.7) 44 (47.3) | 42 371 ± 185 81 to 1031 17 (40.5) 25 (59.5) |
| Serum 1,25 dihydroxy vitamin D (pg/mL) | n Mean ± SD Range Abnormal, n (%) Normal, n (%) | 63 68 ± 25 8 to 166 27 (42.9) 36 (57.1) | 28 67 ± 17 27 to 112 10 (35.7) 18 (64.3) |
| Serum PTH (pmol/L) | n Mean ± SD Range Abnormal, n (%) Normal, n (%) | 88 50 ± 29 5 to 208 13 (14.8) 75 (85.2) | 37 55 ± 28 15 to 123 5 (13.5) 32 (86.5) |
| ALP, alkaline phosphatase; PedsQL, Pediatric Quality of Life Inventory; PTH, parathyroid hormone; SD, standard deviation Abnormal/normal was determined at the site level based on local reference ranges. |  |  |  |

Supplementary Table S2		Variation in PedsQL total, summary and domain scores by bone biochemistry characteristics

| Characteristic |  | n | PedsQL total score |  | Psychosocial Health Summary |  | Physical Health Summary |  | Emotional Functioning |  | Social Functioning |  | School Functioning |  |
| --- | --- | --- | --- | --- | --- | --- | --- | --- | --- | --- | --- | --- | --- | --- |
|  |  |  | Mean or corr. coeff.^a^ | p value | Mean or corr. coeff.^a^ | p value | Mean or corr. coeff.^a^ | p value | Mean or corr. coeff.^a^ | p value | Mean or corr. coeff.^a^ | p value | Mean or corr. coeff.^a^ | p value |
| Serum phosphate | mmol/L | 42 | 0.289 | 0.063 | 0.321 | **0.038** | 0.243 | 0.122 | 0.195 | 0.216 | 0.207 | 0.188 | 0.276 | 0.080 |
|  | Abnormal | 30 | 76.6 | 0.237 | 74.9 | 0.226 | 79.5 | 0.353 | 65.7 | 0.296 | 83.3 | 0.825 | 76.6 | 0.175 |
|  | Normal | 12 | 81.9 |  | 80.6 |  | 84.1 |  | 73.8 |  | 84.6 |  | 83.8 |  |
| Serum 1,25 dihydroxy vitamin D | pg/mL | 28 | 0.001 | 0.998 | −0.041 | 0.836 | 0.093 | 0.637 | −0.167 | 0.397 | −0.022 | 0.910 | 0.136 | 0.498 |
|  | Abnormal | 10 | 75.8 | 0.326 | 73.5 | 0.232 | 79.7 | 0.651 | 59.5 | 0.134 | 83.5 | 0.556 | 79.7 | 0.892 |
|  | Normal | 18 | 81.2 |  | 80.5 |  | 82.5 |  | 73.6 |  | 87.2 |  | 80.5 |  |
| Serum ALP | IU/L | 42 | −0.221 | 0.161 | −0.178 | 0.260 | −0.218 | 0.165 | −0.255 | 0.103 | −0.123 | 0.437 | −0.203 | 0.203 |
|  | Abnormal | 17 | 74.2 | 0.110 | 72.9 | 0.168 | 76.5 | 0.109 | 64.4 | 0.404 | 81.8 | 0.533 | 73.5 | 0.066 |
|  | Normal | 25 | 80.7 |  | 79.0 |  | 83.8 |  | 70.4 |  | 85.0 |  | 82.4 |  |
| Serum PTH | pmol/L | 37 | −0.252 | 0.133 | −0.132 | 0.436 | −0.341 | **0.039** | −0.097 | 0.566 | −0.130 | 0.444 | −0.127 | 0.459 |
|  | Abnormal | 5 | 68.8 | 0.125 | 67.6 | 0.178 | 70.6 | 0.126 | 56.0 | 0.311 | 82.0 | 0.936 | 67.0 | 0.051 |
|  | Normal | 32 | 78.4 |  | 76.6 |  | 81.6 |  | 67.2 |  | 82.7 |  | 80.7 |  |
| Biochemistry characteristics are ± 1 year of PedsQL completion. ^a^Values are mean SF-36 scores for categorical data (e.g. male/female), which were compared using the Mann–Whitney. Spearman’s rank correlation coefficient is reported for ordinal/continuous variables. Significant results (p<0.05) are shown in bold. There are no results for the Physical Functioning domain because it is identical to the Physical Health Summary. ALP, alkaline phosphatase; corr. coeff, correlation coefficient; PedsQL, Pediatric Quality of Life Inventory; PTH, parathyroid hormone |  |  |  |  |  |  |  |  |  |  |  |  |  |  |

**Supplementary Table S3**		**Clinical and surgical history**

| **Characteristic** |  | **n** | **Total score** |  | **Psychosocial Health Summary** |  | **Physical Health Summary** |  | **Emotional Functioning** |  | **Social Functioning** |  | **School Functioning** |  |
| --- | --- | --- | --- | --- | --- | --- | --- | --- | --- | --- | --- | --- | --- | --- |
|  |  |  | **Mean or corr. coeff.** | **p value** | **Mean or corr. coeff.** | **p value** | **Mean or corr. coeff.** | **p value** | **Mean or corr. coeff.** | **p value** | **Mean or corr. coeff.** | **p value** | **Mean or corr. coeff.** | **p value** |
| Nephrocalcinosis | No | 59 | 79.1 | 0.895 | 77.5 | 0.783 | 82.0 | 0.800 | 68.9 | 0.302 | 85.3 | 0.845 | 79.0 | 0.611 |
|  | Yes | 37 | 79.3 |  | 78.2 |  | 81.2 |  | 72.8 |  | 84.1 |  | 77.9 |  |
| Tooth abscess | No | 61 | 79.2 | 0.909 | 77.7 | 0.807 | 81.7 | 0.939 | 70.8 | 0.852 | 84.4 | 0.642 | 78.8 | 0.686 |
|  | Yes | 35 | 79.1 |  | 77.8 |  | 81.5 |  | 69.8 |  | 85.4 |  | 78.2 |  |
| Excessive cavities | No | 74 | 80.2 | 0.234 | 78.9 | 0.197 | 82.6 | 0.492 | 71.7 | 0.367 | 85.7 | 0.336 | 80.0 | 0.162 |
|  | Yes | 22 | 75.5 |  | 73.9 |  | 78.6 |  | 66.0 |  | 81.8 |  | 73.9 |  |
| Genu varum | No | 53 | 81.1 | 0.246 | 80.2 | 0.081 | 82.5 | 0.546 | 72.8 | 0.169 | 86.9 | 0.175 | 81.6 | 0.127 |
|  | Yes | 43 | 76.8 |  | 74.7 |  | 80.7 |  | 67.5 |  | 82.2 |  | 75.0 |  |
| Genu valgum | No | 72 | 78.7 | 0.672 | 77.7 | 0.889 | 80.4 | 0.101 | 68.8 | 0.233 | 85.5 | 0.275 | 79.5 | 0.271 |
|  | Yes | 24 | 80.6 |  | 78.0 |  | 85.4 |  | 75.3 |  | 82.7 |  | 76.0 |  |
| Craniosynostosis | No | 68 | 79.7 | 0.617 | 78.3 | 0.540 | 82.2 | 0.818 | 72.6 | 0.125 | 84.5 | 0.444 | 78.1 | 0.504 |
|  | Yes | 28 | 77.8 |  | 76.4 |  | 80.3 |  | 65.1 |  | 85.5 |  | 79.8 |  |
| Number of corrective surgeries |  | 96 | −0.089 | 0.390 | −0.086 | 0.406 | −0.101 | 0.328 | 0.002 | 0.981 | −0.163 | 0.112 | −0.136 | 0.192 |
| There are too few patients in each group to undertake the analysis for intoeing, tibial torsion, club foot deformity and windswept deformity. Variation in PedsQL scores was assessed using Matt–Whitney (2 categories), Kruskall–Wallis (≥3 categories) and Spearman’s rank correlation coefficient (ordinal/continuous data). None of the results are significant (p<0.05). There are no results for the Physical Functioning domain because it is identical to the Physical Health Summary. |  |  |  |  |  |  |  |  |  |  |  |  |  |  |
